# Supplementary material for: The Association between Physical Environment and Externalising Problems in Typically Developing and Neurodiverse Children and Young People: A Narrative Review
Source: Int J Environ Res Public Health. 2023 Jan 31;20(3):2549. doi: 10.3390/ijerph20032549 (PMC9916018; doi:10.3390/ijerph20032549)
Supplement: Supplementary file 1 [file ijerph-20-02549-s001.zip › ijerph-2080274-supplementary.pdf]

Supplementary Table S1: Search terms (NB: the search terms and strategy were modified for each database searched-all database searches are available from first author)

1. Environment/

2. nature/

3. trees/

4. parks, recreational/

5. natural environment.mp.

6. green space.mp.

7. greenspace.mp.

8. (green and blue space).mp.

9. blue space.mp.

10. bluespace.mp.

11. tree*.mp.

12. (park or parks).mp.

13. or/1-12

14. exp Light/

15. natural light*.mp.

16. ambient light*.mp.

17. environment* light.mp.

18. light pollution.mp.

19. Color/

20. (red and blue).mp.

21. white light.mp.

22. light intensit*.mp.

23. sun light.mp.

24. sunlight.mp.

25. or/14-24

26. exp Sound/

27. exp Noise/

28. Music/

29. noise pollution.mp.

30. (sound scape* or soundscape*).mp.

31. music therap*.mp.

32. urban environment*.mp.

33. urban noise.mp.

34. environment* noise*.mp.

35. neighbo?rhood noise*.mp.

36. or/26-35

37. architecture.mp.

38. biophili*.mp.

39. psychiatric ward design.mp.

40. mental health facilit*.mp.

41. social proximity.mp.

42. personal space.mp.

43. interior design.mp.

44. hospital design.mp.

45. room* layout*.mp.

46. Spatial design.mp.

47. or/37-46

48. exp Air Pollution/

49. exp Temperature/

50. air pollution.mp.

51. air quality.mp.

52. atmospheric pollution.mp.

53. heat.mp.

54. neuroinflammation.mp.

55. traffic emission*.mp.

56. or/48-55

57. exp Externalising behaviours/

58. exp Anger/

59. exp Rage/

60. exp Violence/

61. exp Crime/

62. externali?ing behavio?r*.mp.

63. aggressive behavio?r.mp.

64. challenging behavio?r*.mp.

65. attentional fatigue.mp.

66. psychological restoration.mp.

67. mental fatigue.mp.

68. externalising behaviours.mp.

69. anger.mp.

70. rage.mp.

71. violence.mp.

72. crime.mp.

73. or/57-72

74. 13 or 25 or 36 or 47 or 56

75. 73 and 74

76 exp Child/

77. exp Adolescent/

78. (child* or schoolchild* or preschool* or pre-school* or schoolages* or school-age* or schoolboy* or schoolgirl* or boy* or girl* or preteen* or teen* or adolescent* or youth* or young people or young person* or pediatr* or paediatr*).tw

79. or/76-78

80. 75 and 79

81. exp animals/ not humans.sh.

82. 80 not 81
